# Hospitals and the Spirit Tax

**Published:** 1917-03-03

**Authors:** F. A. Hocking

**Affiliations:** The Dispensary, London Hospital


*To the Editor of* The Hospital.

### Hospitals and the Spirit Tax.

*To the Editor of* The Hospital.

Sir,—May I point out that the writer of the paragraph *re* hospital alcohol is entirely in error in referring to the Government grant to hospitals in relief of the duty paid on alcohol as a "rebate," and further that the entire paragraph is a travesty of the real position?

Hoping you may be able to find room for this brief correction, I am yours faithfully,

F. A. Hocking.

The Dispensary, London Hospital,
February 24, 1917.

*⁎* We publish this letter, but our knowledge of the facts compels us to say that, in the circumstances, and in view of the actual facts known to our correspondent, it is hardly worthy of a president of the Incorporated Association of Hospital Officers. Neither "rebate" nor "relief" is an accurate description, and either might mislead, seeing that the money to be repaid is provided from an annual grant of £10,000 to be made to voluntary hospitals as a compensation for the sums which those hospitals pay for spirit over and above the intrinsic cost of the same. Our correspondent's use of the word "travesty" is grotesque, and but for his knowledge he might have used it in a moment of irritation.—Ed. The Hospital.

